# Correlation of tumor necrosis factor-α and interleukin-1 single-nucleotide polymorphisms with the risk of migraine development

**DOI:** 10.3389/fgene.2025.1556498

**Published:** 2025-04-25

**Authors:** Guijiao Huang, Xin Dong, Xiaojiao Shao, Mengping Wu, Shan Gao, Yixuan Wang, Xinying Guan

**Affiliations:** ^1^ Department of Neurology, The Affiliated Hospital of Kangda College of Nanjing Medical University/ Lianyungang Training Base of Jinzhou Medical University/ The Affiliated Lianyungang Hospital of Xuzhou Medical University, Lianyungang, Jiangsu, China; ^2^ Department of Neurology, The First Affiliated Hospital of Nanjing Medical University, Nanjing, Jiangsu, China; ^3^ Department of Clinical Medicine, The Affiliated Hospital of Kangda College of Nanjing Medical University, Lianyungang, China

**Keywords:** inflammation, interleukins-1, migraine, tumor necrosis factor-alpha, single nucleotide polymorphisms

## Abstract

**Background:**

Migraine, a condition stemming from neurological and vascular irregularities with inflammation involvement, was investigated in a case-control study focusing on the Chinese Han population.

**Methods:**

The research analyzed specific genetic variations—TNF-α −308 G/A, TNF-α −857 C/T, TNF-α −238G/A, IL1B-3953 C/T, and IL1RN −2018T/C SNPs—within 212 migraine patients and 210 healthy controls. Utilizing SNaPshot technology, scientists genotyped these markers related to TNF-α and IL-1 genes.

**Results:**

Findings revealed a significant association between the IL1B−3953 C/T SNP and migraine susceptibility, particularly noting its link with a familial history of the disorder. The presence of the C allele at this location was more prevalent in migraine sufferers. Multivariate analysis reinforced this connection, indicating the C allele under a dominant model as an independent risk factor for migraine (OR = 2.315, 95%CI: 1.053–5.090, P = 0.037). Additionally, the study observed a sex-specific difference regarding the TNF-α −857 C/T SNP among migraine patients.

**Conclusion:**

Overall, this investigation contributes to understanding the genetic underpinnings of migraine in the Han Chinese population, highlighting the IL1B−3953 C/T SNP as a potential biomarker for migraine susceptibility.

## 1 Introduction

Migraine is a common chronic neurological disorder in clinical practice, with a lifetime prevalence of 33% in women and 18% in men (Charles, 2017; MacGregor, 2017; Ferrari et al., 2022). It is a leading cause of disability worldwide and has a profound effect on people living with the disorder, their families, healthcare systems, and society (Saylor and Steiner, 2018). The estimated prevalence of migraine is 15% in the United States of America and 9.3% in China (Burstein et al., 2015). Migraine is recurrent in nature and classically presents as moderate-to-severe head pain lasting 4–72 h. It is typically unilateral with a pulsating quality, accompanied by nausea, vomiting, photophobia, and/or phonophobia, and may be preceded by an aura that consists of optesthesia, sensory, motor, or language symptoms (i.e., migraine with aura (MA)) or may occur without warning [i.e., migraine without aura (MO)] (Charles, 2017; MacGregor, 2017; Ferrari et al., 2022).

The pathophysiology of migraine is not fully understood but likely involves multiple components of the central and peripheral nervous system. Individual migraine attacks are hypothesized to be initiated by complex interactions between genetically determined lowered triggering threshold, fluctuations in endogenous factors that may further lower the triggering threshold, and external trigger factors (Charles, 2018; Cropper and Pradhan, 2022; Ferrari et al., 2022). Still, migraines have significant genetic susceptibility, and their heritability is estimated to be about 50% (Sutherland et al., 2019). The main theories about the pathogenesis of a migraine attack are the vascular theory, the cortical diffusion inhibition theory, the trigeminal vascular theory, and the inflammatory mediators theory (Schulte et al., 2017; Yamanaka et al., 2023). Injurious stimulation of the trigeminal nerve releases a variety of vasoactive peptides, such as calcitonin gene-related peptide (CGRP) and substance P; additionally, they produce inflammatory mediators such as TNF-α and IL-1, causing a cascade that stimulates cerebral vasodilatation, increases the permeability of the vascular wall, and leads to the extravasation of plasma components, mast cell degranulation, release of histamine, and release of inflammatory factors resulting in an aseptic neurogenic inflammatory response, which in turn contributes to the onset of headache symptoms. At the same time, the inflammatory cytokines, such as interleukin (IL) and tumor necrosis factor-α (TNF-α) can induce the hypothalamus to produce prostaglandin E through the nuclear factor κB signaling pathway, which can induce platelet activation caused by platelet-activating factor and thromboxane A2, which cause platelets to release 5-hydroxytryptamine and other vasoactive substances, which in turn affects the metabolism of norepinephrine, etc., ultimately leading to migraine attacks.

Hence, IL-1 and TNF-α are of great research value as important inflammatory mediators involved in migraine attacks (Fawzi et al., 2015). The TNF-α gene cluster is in the highly polymorphic major histocompatibility complex (MHC) class III region on human chromosome 6p21. It comprises four exons and three introns spanning approximately 12 kilobases (kb) (Dong et al., 2012). TNF-α production is influenced by single-nucleotide polymorphisms (SNPs) in the gene’s promoter and is regulated at the transcriptional and post-transcriptional levels. So far, 43 SNPs in the promoter region of the TNF-α gene have been reported in the literature and are involved in a variety of diseases, including Parkinson’s, schizophrenia, cancer, diabetic complications, tuberculosis, and ischemic stroke (Lara-Gómez et al., 2019; Gao et al., 2021; Aytac et al., 2022; Sun et al., 2022). TNF-α SNPs have been associated with migraine risk in Westerner studies (Schurks et al., 2011; Gu et al., 2012; Sudershan et al., 2023), but few studies have been conducted in the Chinese Han population. IL-1β is an active form of IL-1, and the IL1 receptor antagonist (IL1RN) is an IL-1 receptor binding that inhibits the binding of IL-1β to the receptor (Wang et al., 2023). IL1β and IL1RN, as members of the IL1 gene cluster, are located in the segment 2q12-q14 (Mendoza-Carrera et al., 2019). The IL1B−3953C/T (rs1143634) and IL1RN −2018T/C (rs419598) SNPs are involved in a variety of diseases, such as rheumatoid arthritis, multiple sclerosis, and periodontitis (Asgharzadeh et al., 2020; Gomes da Silva et al., 2020; Brodzikowska et al., 2022). Still, few studies have examined IL-1 SNPs and migraines.

Therefore, this case-control study explored the associations of the TNF-α −308 G/A (rs1800629), TNF-α −857 C/T (rs1799724), TNF-α −238G/A (rs361525), IL1B-3953 C/T (rs1143634), and IL1RN −2018T/C (rs419598) SNPs with migraine in the Chinese Han population. This study aims to establish a foundation for potential targeted therapies in the future.

## 2 Materials and Methods

### 2.1 Study design and population

This case-control study enrolled patients with migraine attending the outpatient clinic of the Department of Neurology of The First People’s Hospital of Lianyungang, Jiangsu Province, China, and sex- and age-matched healthy individuals from the medical examination center of the same hospital. The study was approved by the ethics committee of The First People’s Hospital of Lianyungang (approval # LW20171231001/LW20171231002). All participants provided written informed consent before any study procedure.

The inclusion criteria were 1) patient admitted at the neurology outpatient clinic and 2) diagnosis of migraine based on magnetic resonance imaging (MRI), rigorous neurological examination, computed tomography (CT), and detailed questionnaires according to ICHD-II criteria. The exclusion criteria were 1) psychiatric disorders, 2) cardiovascular or cerebrovascular diseases, cancer, asthma, anxiety, or depression, or 3) migraine secondary to another disorder. Healthy individuals with headaches were recruited from the Physical Examination Department as controls. They were matched for age (±5 years) and sex with the patients with migraine. The exclusion criteria were the same as for the patients. All participants were unrelated and Han Chinese.

### 2.2 Determination of the single nucleotide polymorphisms

Peripheral venous blood (2 mL) was collected from all participants in EDTA-K_2_ tubes and stored at −80°C. The samples were processed in batches. The blood was thawed at room temperature, and DNA was extracted using the TIANGEN Blood Genomic DNA Extraction Kit (Shanghai Jierui Bioengineering Co., Ltd.) according to the manufacturer’s instructions. The primers (Table 1) were synthesized by Shanghai Jierui Bioengineering Co., Ltd. (Shanghai, China). After amplification, 1 µL of the extension product was added to 9 µL of HIDI, denatured at 95°C for 3 min, immediately immersed in ice water, and loaded on the SNaPshot system. The sample’s genotype was determined according to the color of the peak, and the corresponding SNP site of the extension product was determined according to the position of the peak. Some samples were selected for validation by Sanger sequencing (Supplementary Figures S1–S5).

**TABLE 1:** Primers used for PCR.

SNP	Sense	Sequence (5’->3′)
rs1800629	Forward	TTT​CTC​TCC​CTC​AAG​GAC​TC
	Reverse	TTC​TGT​CTC​GGT​TTC​TTC​TC
	Extension	GCA​ATA​GGT​TTT​GAG​GGG​CAT​G
rs1799724	Forward	GAA​GCT​GAG​AAG​ATG​AAG​GA
	Reverse	GGA​GAC​TCA​TAA​TGC​TGG​TT
	Extension	TTT​TTT​TTT​TCC​CTC​TAC​ATG​GCC​CTG​TCT​TC
rs361525	Forward	TTT​CTC​TCC​CTC​AAG​GAC​TC
	Reverse	TTC​TGT​CTC​GGT​TTC​TTC​TC
	Extension	ACA​CTC​CCC​ATC​CTC​CCT​GCT​C
rs1143634	Forward	TAC​CTT​GTT​GCT​CCA​TAT​CC
	Reverse	CTA​CTG​GTG​TTG​TCA​TCA​GA
	Extension	TTT​TTT​TTT​TTT​TTT​TTT​AAG​CCT​CGT​TAT​CCC​ATG​TGT​C
rs419598	Forward	AAT​ACT​ATA​CCC​CTG​GAA​GAG
	Reverse	CCA​ACA​AGG​ATT​AGG​ACA​TT
	Extension	CAT​TTG​GTC​CTT​GCA​AGT​ATC​C

### 2.3 Sample size

According to the relevant literature, the incidence of SNPs in migraine patients and healthy people was estimated at p_A_ = 91.7% and p_B_ = 79.3%, respectively. Using the equation below and α = 0.05, κ = 1, β = 0.9, z1−α, and z1−β (the standard normal distribution quantile expressed in phase with α and β values, respectively), the minimal sample size for each group was determined as 140. Considering a possible loss of 10% due to consent withdrawal or technical failure, 154 cases were needed for each group.

$$n=\left(\frac{p_{A}\left(1-p_{A}\right)}{\kappa }+p_{B}\left(1-p_{B}\right)\right){\left(\frac{z_{1-\alpha }+z_{1-\beta }}{p_{A}-p_{B}}\right)}^{2}$$

### 2.4 Statistical analysis

The results were analyzed using SPSS 26.0 (IBM, Armonk, NY, United States). The continuous data were expressed as medians (25th and 75th percentile). Age comparisons were made using the independent samples t-test. Categorical data were expressed as n (%). The comparisons of genotypes and alleles between groups and strata were made using the chi-square test or Fisher’s exact test. Hardy-Weinberg’s law of equilibrium was used to assess whether the samples complied with the law of genetic equilibrium. Univariable and multivariable logistic regression analyses were used to examine the associations of the SNPs with migraine. The results were presented as odds ratios (ORs) and 95% confidence intervals (CIs). Two-sided P-values <0.05 were considered statistically significant.

A multivariable logistic regression model was constructed to examine the relationship between SNPs and migraine susceptibility. Adjustments were made for migraine type (MO vs MA), family history of migraine, age, and sex, based on their potential biological relevance and previous studies (Dong et al., 2012; Fawzi et al., 2015; Khosravi et al., 2015; Dahash and Kusrat Hussein, 2023). The model was fitted using a step-by-step selection process to minimize overfitting and ensure robustness.

Additionally, a multifactor dimensionality reduction (MDR) analysis was conducted to address gene-gene and gene-environment interactions. MDR aggregates multi-locus genotypes into “high-risk” and “low-risk” categories, reducing dimensionality from N to a single dimension. Cross-validation and permutation testing were used to assess the predictive efficacy of the derived variables.

## 3 Results

### 3.1 Characteristics of the participants

The study enrolled 422 participants: 212 patients with migraine and 210 healthy controls. There were no statistically significant differences in age (P = 0.213) and sex (P = 0.783) (Table 2).

**TABLE 2:** Demographic characteristics.

Parameters	Control group	Case group	P
Age	33 (26,41)	30 (25,40)	0.213
Sex (%)			
Male	74 (35.2)	72 (34.0)	0.783
Female	136 (64.8)	140 (66.0)	
MA/MO		30/182	
Family history (no/yes)		105/107	

MA: migraine with aura; MO: migraine without aura.

### 3.2 Hardy-weinberg equilibrium

The genotypes and alleles of the included SNPs were tested using the Hardy-Weinberg balanced test (Supplementary Table S1), and all conformed to Mendel’s law of inheritance (all P > 0.05).

Genotype and allele associations of controls with migraine and migraine subtypesThe IL1B−3953 C/T (rs1143634) genotype was associated with migraine (P = 0.038) and with a family history of migraine (P = 0.050). The C allele frequency was higher in the migraine group than in the control group (P = 0.041). Based on the dominant model, the C allele was associated with migraine (P = 0.038) and with a family history of migraine (P = 0.050). The other SNPs analyzed in this study were not associated with migraine, migraine type, or a family history of migraine (all P > 0.05) (Table 3). The multivariable analysis showed that after adjustment for migraine type (MA/MO), family history, age, and sex, the C allele of the IL1B−3953 C/T (rs1143634) locus (dominant model) was independently associated with migraine (OR = 2.315, 95%CI: 1.053–5.090, P = 0.037) (Table 4).

**TABLE 3:** Analysis of genotypes and alleles at different loci in the control and migraine groups and subtypes.

SNPs	Controls (n = 210)	Cases (n = 216)	P Case vs control	MA (n = 30)	P MA vs control	MO (n = 182)	P MO vs control	Family history (n = 107)	P History vs control	No family history (n = 105)	P No history vs control
Rs1800629			0.553		>0.999		0.627		0.882		0.881
GG	178 (84.8)	180 (84.9)		26 (86.7)		154 (84.6)		91 (85.0)		89 (84.8)	
GA	30 (14.3)	32 (15.1)		4 (13.3)		28 (15.4)		16 (15.0)		16 (15.2)	
AA	2 (1.0)	0		0		0		0		0	
G	386 (91.9)	376 (92.5)	0.767	56 (93.3)	0.898	336 (92.3)	0.835	198 (92.5)	0.785	194 (92.4)	0.835
A	34 (8.1)	32 (7.5)		4 (6.7)		28 (7.7)		16 (7.5)		16 (7.6)	
GA + AA/GG	32/178	32/180	0.967	4/26	>0.999	28/154	0.968	16/91	0.947	16/89	>0.999
Rs1799724			0.550		0.384		0.808		0.890		0.394
CC	157 (74.8)	166 (78.3)		26 (86.7)		140 (76.9)		83 (77.6)		83 (79.0)	
CT	49 (23.3)	44 (20.8)		4 (13.3)		40 (22.0)		22 (20.6)		22 (20.6)	
TT	4 (1.9)	2 (0.9)		0		2 (1.1)		2 (1.9)		0	
C	363 (86.4)	376 (88.7)	0.322	56 (93.3)	0.133	320 (87.9)	0.536	188 (87.9)	0.616	188 (89.5)	0.269
T	57 (13.6)	48 (11.3)		4 (6.7)		44 (12.1)		26 (12.1)		22 (10.5)	
CT + TT/CC	53/157	46/166	0.391	4/26	0.152	42/140	0.618	24/83	0.581	22/83	0.400
Rs361525			0.922		0.337		0.910		>0.999		0.759
GG	196 (93.3)	198 (93.4)		26 (86.7)		172 (94.5)		101 (94.4)		97 (92.4)	
GA	13 (6.2)	14 (6.6)		4 (13.3)		10 (5.5)		6 (5.6)		8 (7.6)	
AA	1 (0.5)	0		0		0		0		0	
G	405 (96.4)	410 (96.7)	0.830	56 (93.3)	0.426	354 (97.3)	0.512	208 (97.2)	0.610	202 (96.2)	0.881
A	15 (3.6)	14 (3.3)		4 (6.7)		10 (2.7)		6 (2.8)		8 (3.8)	
GA + AA/GG	14/196	14/198	0.979	4/26	0.354	10/172	0.629	6/101	0.714	8/97	0.755
Rs1143634			0.038*		0.398		0.060		0.050*		0.200
CC	189 (90.0)	202 (95.3)		29 (96.7)		173 (95.1)		103 (96.3)		99 (94.3)	
CT	21 (10.0)	10 (4.7)		1 (3.3)		9 (4.9)		4 (3.7)		6 (5.7)	
TT	0	0		0		0		0		0	
C	399 (95.0)	414 (97.6)	0.041*	59 (98.3)	0.409	355 (94.9)	0.959	210 (98.1)	0.055	204 (97.1)	0.211
T	21 (5.0)	10 (2.4)		1 (1.7)		19 (5.1)		4 (1.9)		6 (2.9)	
CT + TT/CC	21/189	10/202	0.038*	1/29	0.398	9/173	0.060	4/103	0.050*	6/99	0.200
Rs419598			0.931		>0.999		>0.999		0.697		0.667
TT	191 (91.0)	194 (91.5)		28 (93.3)		166 (91.2)		95 (88.8)		99 (94.3)	
CT	18 (8.6)	18 (8.5)		2 (6.7)		16 (8.8)		12 (11.2)		6 (5.7)	
CC	1 (0.5)	0		0		0		0		0	
T	400 (95.2)	406 (95.8)	0.717	58 (96.7)	0.869	348 (95.6)	0.807	202 (94.4)	0.646	204 (97.1)	0.257
C	20 (4.8)	18 (4.2)		2 (3.3)		16 (4.4)		12 (5.6)		6 (2.9)	
CT + CC/TT	19/191	18/194	0.840	2/28	0.931	16/166	0.929	12/95	0.539	6/90	0.302

MA: migraine with aura; MO: migraine without aura.

**TABLE 4:** Univariable and multivariable analyses of the polymorphic loci with migraine susceptibility.

SNPs	Dominant model	Univariable OR	P	Multivariable OR	P
TNF-α-308 G/A (rs1800629)	GA + AA/GG	1.011 (0.594–1.721)	0.967	0.978 (0.573–1.671)	0.935
TNF-α-857 C/T (rs1799724)	CT + TT/CC	1.218 (0.776–1.913)	0.391	1.219 (0.771–1.928)	0.396
TNF-α-238G/A (rs361525)	GA + AA/GG	1.010 (0.469–2.175)	0.979	1.018 (0.472–2.195)	0.963
IL1B-3953 C/T (rs1143634)	CT + TT/CC	2.244 (1.030–4.890)	0.042*	2.315 (1.053–5.090)	0.037*
IL1RN −2018 T/C (rs419598)	CT + CC/TT	1.072 (0.546–2.106)	0.840	1.133 (0.573–2.239)	0.719

OR: odds ratio; CI: confidence interval.

The multivariable analysis was adjusted for migraine type (MA/MO), family history, age, and sex. The * symbol represents P s 0.05, which is statistically significant.

### 3.3 Multifactor dimensionality reduction

**FIGURE 1:**
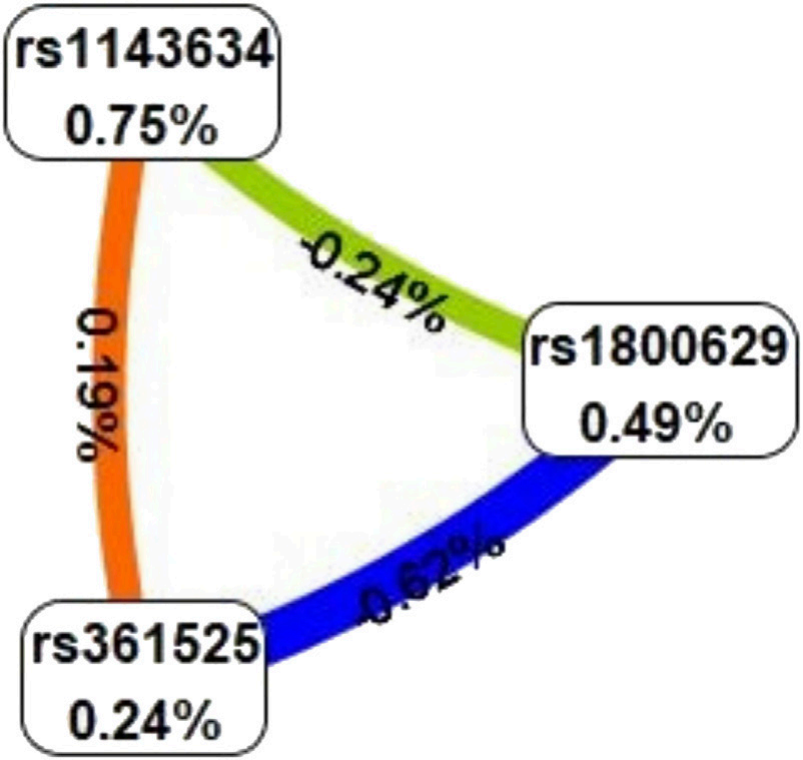
High order interaction entropy diagram of genes. The color gradient corresponds to the entropy of information gain, spanning from red (denoting the maximum information gain) through yellow-green to blue (representing the greatest information redundancy).

**FIGURE 2:**
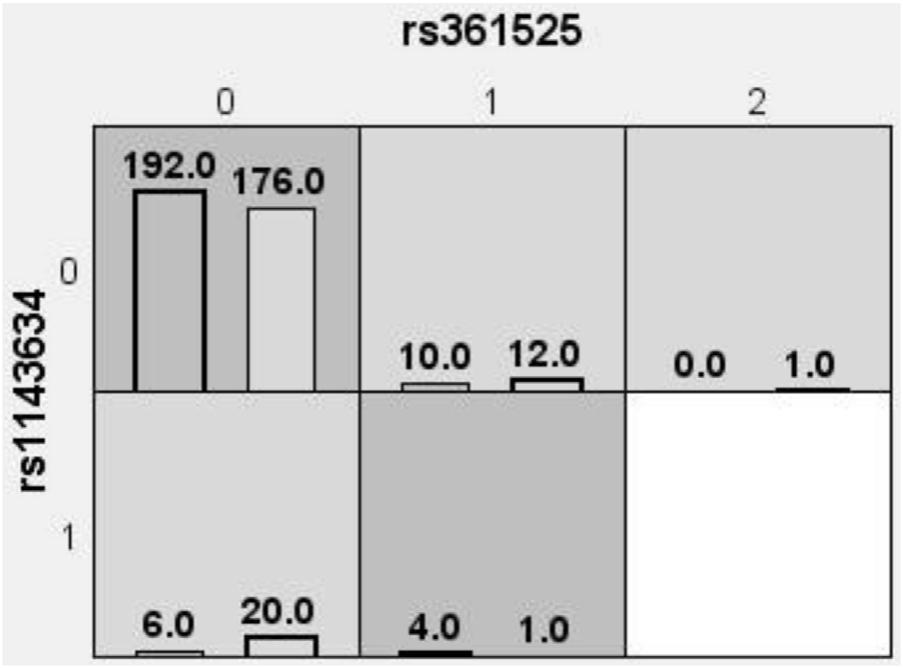
High-risk and low-risk genotypes within the optimal interaction model. Dark gray cells signify high risk (with a case-to-control ratio of the genotype combination ≥1.0), whereas light Gy cells denote low risk (with a case-to-control ratio of the genotype combination ≤1.0). 0 = wild-type homozygote, 1 = heterozygote, 2 = mutant homozygote.

The optimal interaction model identified is the second-order interaction between rs361525 and rs1143634. This model demonstrates the highest validation accuracy of 50.07% and a cross-validation consistency of 8 out of 10. The linkage coefficient between rs1143634 and rs361525 is 0.19% (Table 5). Figure 1 illustrates the high-order interaction entropy among three genetic loci: rs1143634, rs1800629, and rs361525. The information gain entropy of rs1143634 is the highest (0.75%), indicating its greatest contribution to disease prediction, while rs361525 has the smallest contribution (0.24%). Figure 2 displays high-risk and low-risk genotypes within the optimal interaction model. Dark gray cells indicate high risk (case-to-control ratio of the genotype combination ≥1.0), while light Gy cells denote low risk (case-to-control ratio of the genotype combination ≤1.0). In the figure, the combination of rs361525 = 0 and rs1143634 = 0 (case-to-control ratio = 192.0) exhibits the highest risk, whereas the combination of rs361525 = 2 and rs1143634 = 0 (case-to-control ratio = 0.0) shows the lowest risk. These results reveal key genetic loci and their interactions, providing important clues for disease prediction and mechanistic research.

**TABLE 5:** Multifactor interaction model for MDR analysis of influencing factors of migraine in population.

Models	Training sample accuracy	Testing sample accuracy	Cross-validation consistency
rs1143634	0.5274	0.4911	7/10
rs361525, rs1143634	0.5423	0.5007	8/10
rs1800629, rs361525, rs1143634	0.551	0.4726	6/10

### 3.4 Associations of genotypes and alleles with patient characteristics among patients with migraine

Among the patients with migraine, significant differences between sexes were observed at the TNF-α −857 C/T (rs1799724) locus at the genotype (P = 0.016), T allele frequency (P = 0.013), and T allele in the dominant model (P = 0.025), with the T alleles being more common in males. No other associations were observed (all P > 0.05) (Table 6).

**TABLE 6:** Results of the stratified analysis of migraine sufferers.

SNPs	MA (n = 30)	MO (n = 182)	P MA vs MO	Family history (n = 107)	No family history (n = 105)	P With vs without history	Female (n = 140)	Male (n = 72)	P Male vs female
Rs1800629			0.988			0.954			0.647
GG	26 (86.7)	154 (84.6)		91 (85.0)	89 (84.8)		120 (85.7)	60 (83.3)	
GA	4 (13.3)	28 (15.4)		16 (15.0)	16 (15.2)		20 (14.3)	12 (16.7)	
AA	0	0		0	0		0	0	
G	56 (93.3)	336 (92.3)	0.988	198 (92.5)	194 (92.4)	0.956	260 (92.9)	132 (91.7)	0.660
A	4 (6.7)	28 (7.7)		16 (7.5)	16 (7.6)		20 (7.1)	12 (8.3)	
GA + AA/GG	4/26	28/154	0.988	16/91	16/89	0.954	20/120	12/60	0.647
Rs1799724			0.513			0.616			0.016*
CC	26 (86.7)	140 (76.9)		83 (77.6)	83 (79.0)		116 (82.9)	50 (69.4)	
CT	4 (13.3)	40 (22.0)		22 (20.6)	22 (20.6)		24 (17.1)	20 (27.8)	
TT	0	2 (1.1)		2 (1.9)	0		0	2 (2.8)	
C	56 (93.3)	320 (87.9)	0.219	188 (87.9)	188 (89.5)	0.587	256 (91.4)	120 (83.3)	0.013*
T	4 (6.7)	44 (12.1)		26 (12.1)	22 (10.5)		24 (8.6)	24 (16.7)	
CT + TT/CC	4/26	42/140	0.230	24/83	22/83	0.794	24/116	22/50	0.025*
Rs361525			0.228			0.555			0.663
GG	26 (86.7)	172 (94.5)		101 (94.4)	97 (92.4)		132 (94.3)	66 (91.7)	
GA	4 (13.3)	10 (5.5)		6 (5.6)	8 (7.6)		8 (5.7)	6 (8.3)	
AA	0	0		0	0		0	0	
G	56 (93.3)	354 (97.3)	0.236	208 (97.2)	202 (96.2)	0.562	272 (97.1)	138 (95.8)	0.669
A	4 (6.7)	10 (2.7)		6 (2.8)	8 (3.8)		8 (2.9)	6 (4.2)	
GA + AA/GG	4/26	10/172	0.228	6/101	8/97	0.555	8/132	6/66	0.663
Rs1143634			>0.999			0.723			0.195
CC	29 (96.7)	173 (95.1)		103 (96.3)	99 (94.3)		131 (93.6)	71 (98.6)	
CT	1 (3.3)	9 (4.9)		4 (3.7)	6 (5.7)		9 (6.4)	1 (1.4)	
TT	0	0		0	0		0	0	
C	59 (98.3)	355 (94.9)	0.401	210 (98.1)	204 (97.1)	0.726	271 (96.8)	143 (99.3)	0.200
T	1 (1.7)	19 (5.1)		4 (1.9)	6 (2.9)		9 (3.2)	1 (0.7)	
CT + TT/CC	1/29	9/173	>0.999	4/103	6/99	0.723	9/131	1/71	0.195
Rs419598			0.973			0.151			0.272
TT	28 (93.3)	166 (91.2)		95 (88.8)	99 (94.3)		126 (90.0)	68 (94.4)	
CT	2 (6.7)	16 (8.8)		12 (11.2)	6 (5.7)		14 (10.0)	4 (5.6)	
CC	0	0		0	0		0	0	
T	58 (96.7)	348 (95.6)	0.974	202 (94.4)	204 (97.1)	0.160	266 (95.0)	140 (97.2)	0.282
C	2 (3.3)	16 (4.4)		12 (5.6)	6 (2.9)		14 (5.0)	4 (2.8)	
CT + CC/TT	2/28	16/166	0.973	12/95	6/99	0.151	14/126	4/68	0.272

MA: migraine with aura; MO: migraine without aura.

## 4 Discussion

Migraine is a disease caused by neurovascular dysfunction, and inflammatory factors play an important role in migraine pathogenesis. This case-control study explored the associations of SNPs with migraine in the Chinese Han population. The results showed that the IL1B−3953 C/T (rs1143634) SNP is associated with susceptibility to migraine in Han Chinese. These results could have significance in managing patients with migraine and their families.

IL1B−3953 C/T (rs1143634) is in the exon five coding region, and the nucleotide change from C to T causes a synonymous mutation. Nevertheless, this study found that the frequency of the C allele in the migraine group was significantly higher than in the healthy control group, and the CT + TT genotype has a certain protective effect on migraine in the Han population. On the other hand, in a previous study in the United States, Yilmaz et al. (2010) found that the IL1B−3953 T allele was higher in migraine patients than in healthy controls. This discrepancy was also observed for other diseases among different populations. In the Iraqi population, it was shown that the TT genotype was significantly reduced in periodontitis cases (Dahash and Kusrat Hussein, 2023), while that association was not observed in the Polish population (Brodzikowska et al., 2022). In Brazil, the C/T genotype was reported to have a protective effect on rheumatoid arthritis (Gomes da Silva et al., 2020), while no differences in distribution between rheumatoid patients and controls were found in the Algerian population (Allam et al., 2013). The IL1B+3953 T allele and T/T genotype increase the risk of multiple sclerosis in Iranians (Khosravi et al., 2015). Another study in the Iranian Azerbaijani population found no significant correlation between the multiple system sclerosis group and the control group (Asgharzadeh et al., 2020). Hence, the IL1B−3,953 (rs1143634) SNP shows different effects in different diseases or across different populations. The IL-1 family members are first synthesized into inactive precursors that cannot bind to their receptors and then activated by the inflammasome (Dinarello, 2018). There are two types of IL-1 receptors in humans (Dinarello, 2018; Mailhot et al., 2020). Studies have shown that IL-1R1 ligation can lead to a variety of inflammatory responses, while IL-1R2 has also been shown to be a decoy receptor that provides a negative regulatory mechanism for inflammation (Peters et al., 2013). Therefore, different SNPs and mutations may also lead to selective binding to different receptors and effects. The allele C of Il-1β changed into allele T, which changed the ability of its competitive binding with IL-1RN to the IL-1 receptor, broke the balance between anti-inflammatory and pro-inflammatory, and led to the difference in disease among people (Tahmasebi et al., 2013). The scope of the heterogeneity of IL1B−3,953 (rs1143634) in the worldwide population is still unknown, but the present study strongly suggests that IL1B−3,953 (rs1143634) is associated with migraine in the Han Chinese population. On the other hand, the stratified analysis showed no statistically significant sex-specific differences in IL1B-3953 C/T polymorphisms in migraine patients (P = 0.195). However, the IL1B-3953 C/T SNP was significantly associated with total migraine susceptibility in the Han population. Therefore, although this SNP is associated with migraine risk, its effect does not appear to be dependent on sex in the study population.

The present study did not find significant differences in the three SNPs in the promoter region of TNF-α between migraine and control subjects. However, the differences of TNF-α −308G/A (rs1800629) and −857 (rs1799724) between migraine and control subjects were previously found in the Jordanian (Hamad et al., 2021) and Egyptian populations (Fawzi et al., 2015), which may be related to the heterogeneity of migraine. On the other hand, the present study showed sex differences in the genotype, allele, and dominant model of the TNF-α −857 (rs1799724) polymorphism in migraine patients. Since there is an important difference in migraine prevalence between men and women (Charles, 2017; MacGregor, 2017; Ferrari et al., 2022; Zhang and Robbins, 2023), the TNF-α −857 (rs1799724) genotype could play a role. However, this present study did not control the age of the enrolled patients. A Greek study in children (Pappa et al., 2010) found a significant difference in the genotype, allele, and dominant model of the TNF-α −857 (rs1799724) polymorphism in migraine patients. The difference in the results may be related to estrogen content because the difference between men and women is small before puberty. Still, the content of estrogen in women increases after puberty and peaks in the reproductive age, consistent with the peak of migraine attack frequency in the young and middle-aged and decreases after menopause (Nappi et al., 2022). It is also possible that the immune inflammatory mechanism underlying migraine has not been formed in childhood, thus affecting the expression of TNF-α (Boćkowski et al., 2010). The multivariable logistic regression analysis showed no significant associations between the TNF-α-857 C/T SNP and migraine risk, but sex-specific differences were observed in the distribution of the TNF-α-857 C/T SNP in migraine patients. Specifically, the T allele was more prevalent in males. Although this SNP does not directly increase overall migraine risk, its sex-specific distribution suggests a potential role in the differential migraine mechanisms between sexes.

The analysis showed no significant differences in genotype or allele frequencies between MA and MO for TNF-α-857 C/T (rs1799724) or IL1B−3953 C/T (rs1143634) (all P > 0.05). Therefore, these SNPs do not appear to affect migraine subtype differentiation in the study population.

This study has limitations. First, migraine, except for familial hemiplegic migraine that is caused by a single gene, is mostly considered to be a polygenic multifactorial disease, and the results of the associations will vary among different populations with different genetics and environmental exposures. Hence, future studies on migraine will have to consider environmental and genetic factors. In this paper, only the local population was included, limiting generalizability.

The IL1B−3953 C/T (rs1143634) SNP is associated with migraine susceptibility in the Han Chinese population. There are sex differences at the TNF-α −857 C/T (rs1799724) locus in migraine patients. No significant associations were found between TNF-α −308 G/A (rs1800629), TNF-α −857 C/T (rs1799724), TNF-α −238G/A (rs361525), and IL1RN −2018 T/C (rs419598) and migraine.

## Data Availability

The original contributions presented in the study are included in the article/Supplementary Material, further inquiries can be directed to the corresponding author/s.

## Supplementary material

SUPPLEMENTARY FIGURE S1 Sanger sequencing confirmation of rs1800629.GAGGCAATAGGTTTTGAGGGGCATG[A/G]GGACGGGGTTCAGCCTCCAGGGTCC.

SUPPLEMENTARY FIGURE S2 Sanger sequencing confirmation of rs1799724. GTC​GAG​TAT​GGG​GAC​CCC​CCC​TTA​A[C/T]GAA​GAC​AGG​GCC​ATG​TAG​AGG​GCC​C.

SUPPLEMENTARY FIGURE S3 Sanger sequencing confirmation of rs361525. GGC​CCA​GAA​GAC​CCC​CCT​CGG​AAT​C[A/G]GAG​CAG​GGA​GGA​TGG​GGA​GTG​TGA​G.

SUPPLEMENTARY FIGURE S4 Sanger sequencing confirmation of rs1143634. CAT​AAG​CCT​CGT​TAT​CCC​ATG​TGT​C[A/G]AAG​AAG​ATA​GGT​TCT​GAA​ATG​TGG​A.

SUPPLEMENTARY FIGURE S5 Sanger sequencing confirmation of rs419598. ATC​TGA​GGA​ACA​ACC​AAC​TAG​TTG​C[C/T]GGA​TAC​TTG​CAA​GGA​CCA​AAT​GTC​A.
