# Supplementary figures and images for: Correlation of tumor necrosis factor-α and interleukin-1 single-nucleotide polymorphisms with the risk of migraine development
Source: Front Genet. 2025 Apr 25;16:1556498. doi: 10.3389/fgene.2025.1556498 (PMC12061721; doi:10.3389/fgene.2025.1556498)

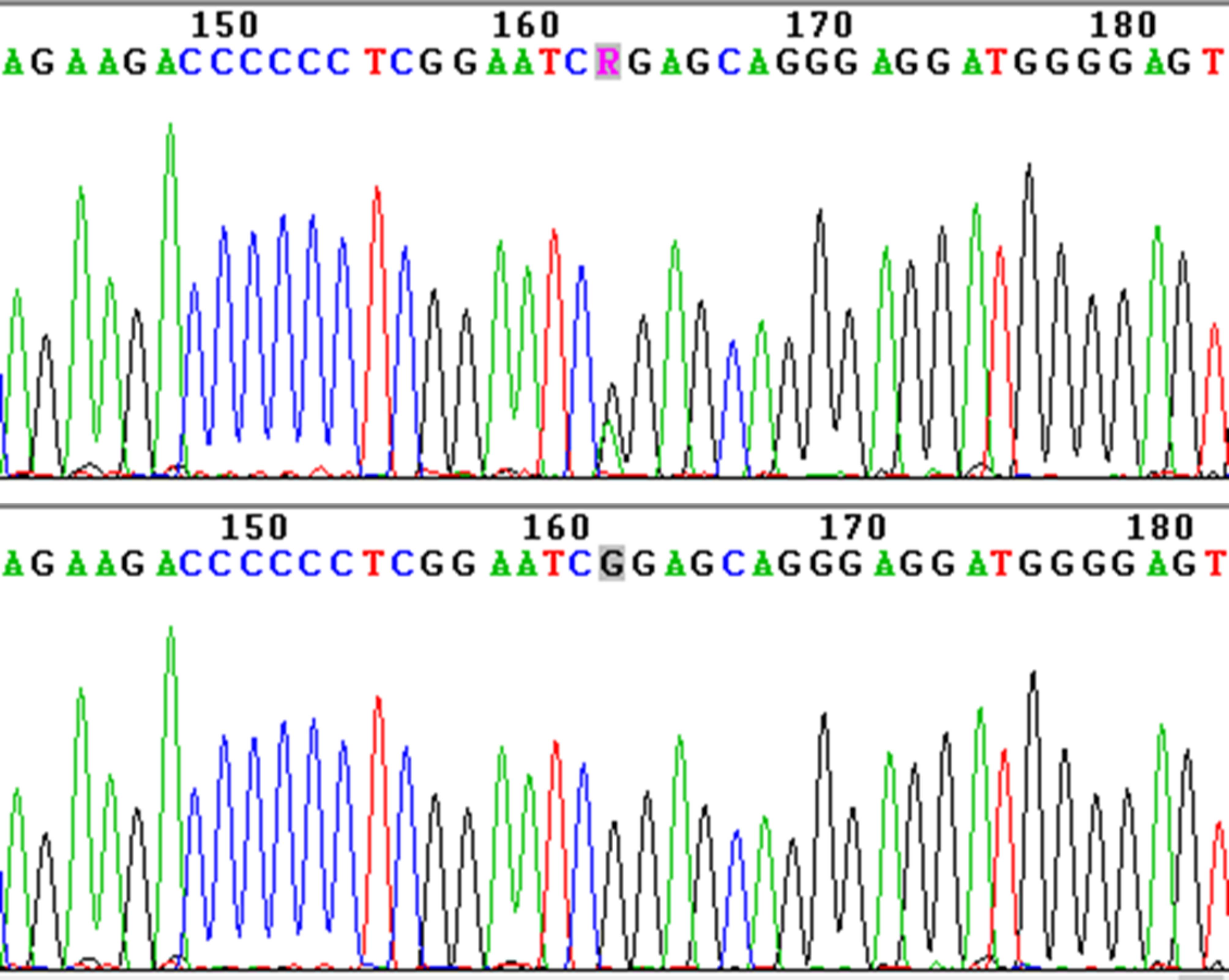

Supplement: Supplementary file 1 [file Image3.JPEG]

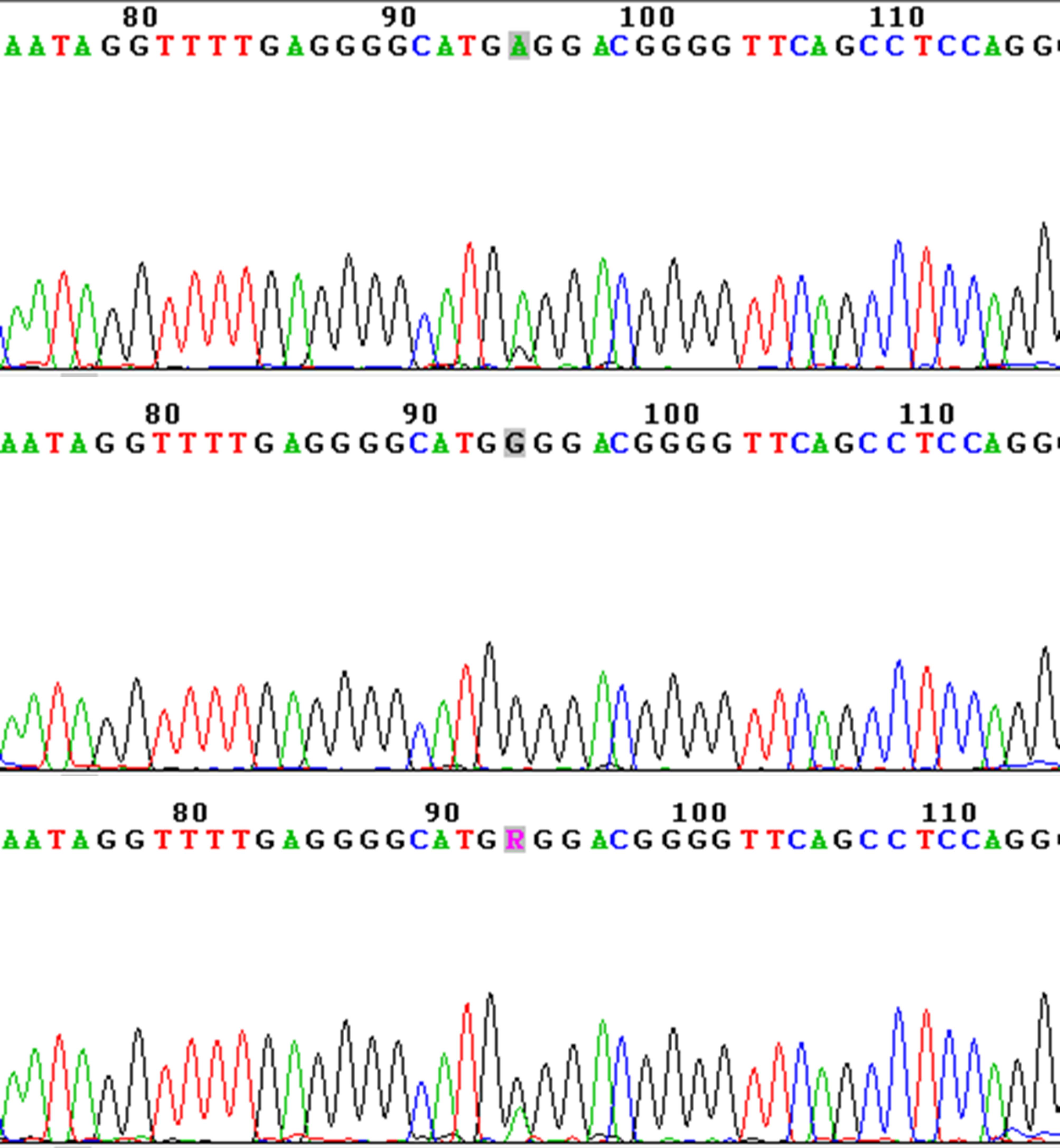

Supplement: Supplementary file 3 [file Image1.JPEG]

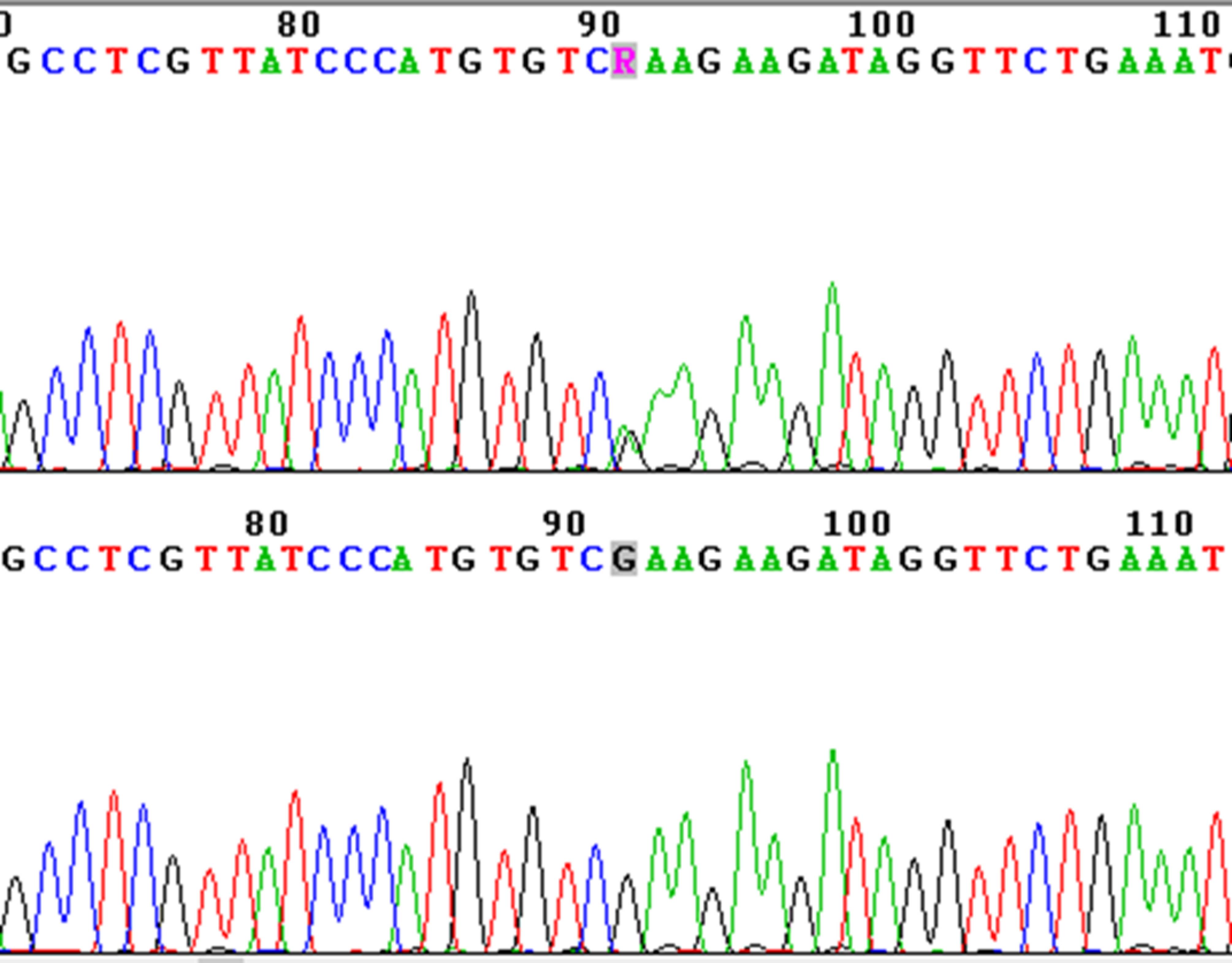

Supplement: Supplementary file 4 [file Image4.JPEG]

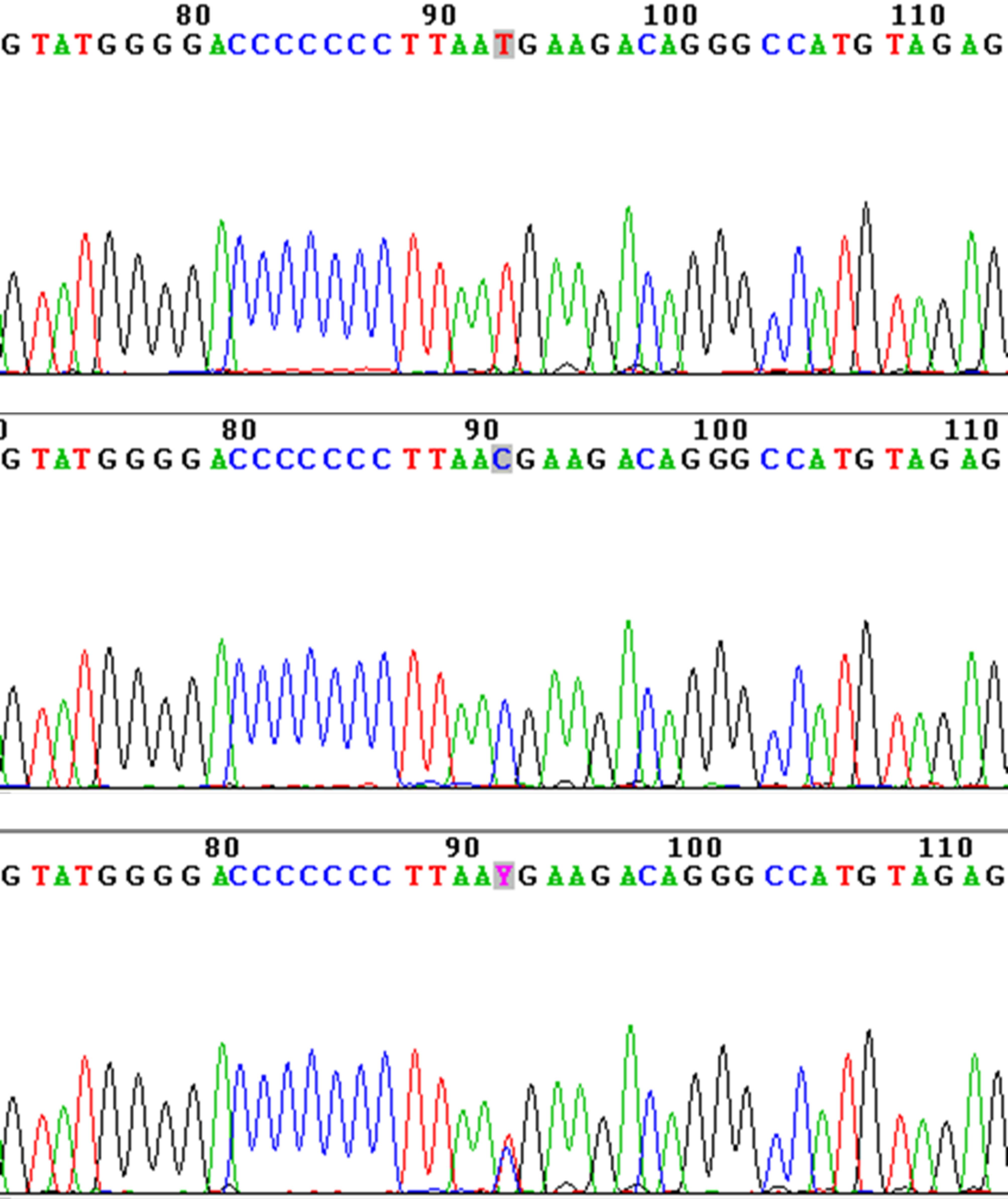

Supplement: Supplementary file 5 [file Image2.JPEG]

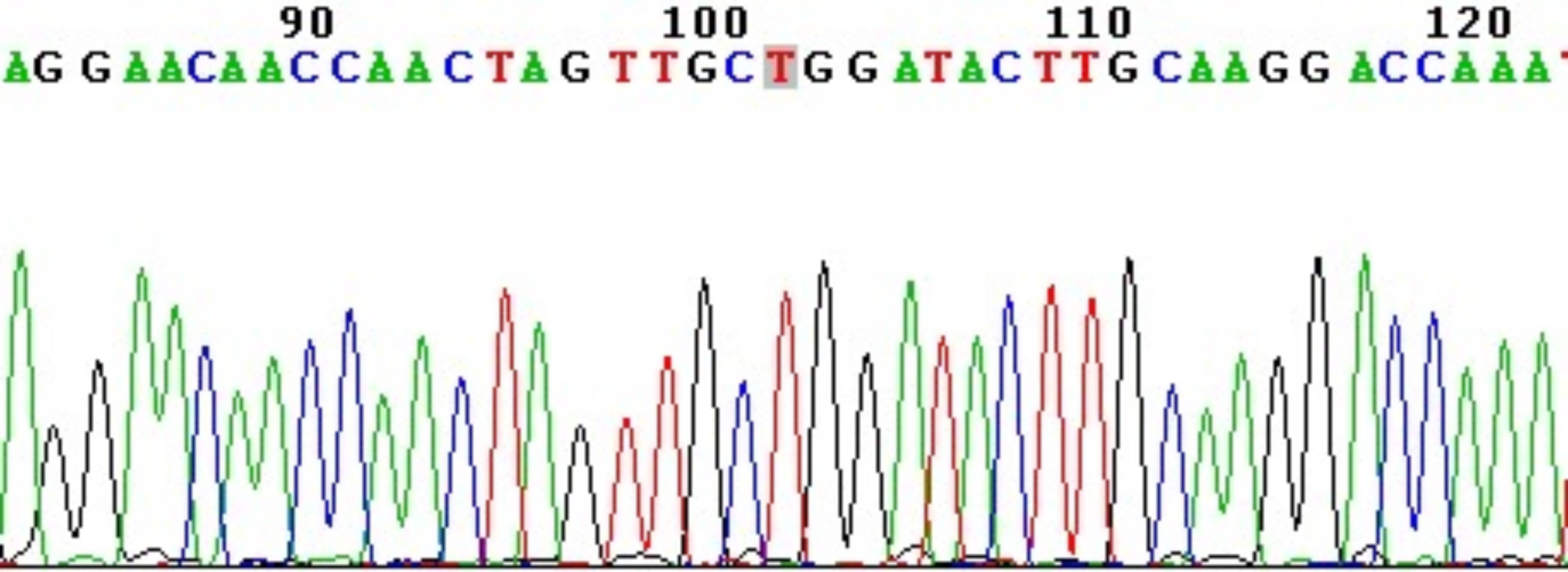

Supplement: Supplementary file 6 [file Image5.JPEG]
