# Supplementary material for: Correlation of tumor necrosis factor-α and interleukin-1 single-nucleotide polymorphisms with the risk of migraine development
Source: Front Genet. 2025 Apr 25;16:1556498. doi: 10.3389/fgene.2025.1556498 (PMC12061721; doi:10.3389/fgene.2025.1556498)
Supplement: Supplementary file 2 [file Table1.DOCX]

**Supplementary Table S1.** Results of the Hardy-Weinberg Equilibrium test

| SNPs | P for HWE (controls) | P for HWE (cases) |
| --- | --- | --- |
| TNF-α -308 G/A (rs1800629 | 0.563 | 0.235 |
| TNF-α -857 C/T (rs1799724) | 0.938 | 0.624 |
| TNF-α -238G/A (rs361525) | 0.142 | 0.619 |
| IL1B -3953 C/T (rs1143634) | 0.446 | 0.725 |
| IL1RN -2018 T/C (rs419598) | 0.425 | 0.519 |

HWE: Hardy-Weinberg equilibrium
